# Effect of Solder Layer Void Damage on the Temperature of IGBT Modules

**DOI:** 10.3390/mi14071344

**Published:** 2023-06-30

**Authors:** Pengpeng Xu, Peisheng Liu, Lei Yan, Zhao Zhang

**Affiliations:** Jiangsu Key Laboratory of ASIC Design, Nantong University, Nantong 226019, China

**Keywords:** void, maximum junction temperature, IGBT module, ANSYS finite element analysis, reliability

## Abstract

Solder layer void is one of the main failure causes of power semiconductor devices, which will seriously affect the reliability of the devices. In this study, a 3D model of IGBT (Insulated Gate Bipolar Transistor) packaging was built by DesignModeler. Based on ANSYS Workbench, the influence of void size, location, solder layer type, and thickness on the temperature distribution of the IGBT module was simulated. The results show that the larger the void radius, the higher the temperature of the IGBT module. The closer the void is to the center of the solder layer, the higher the temperature of the module. The void on the top corner of the solder layer had the greatest impact on the junction temperature of the IGBT module, and the shape of the void is also one of the factors that affect the temperature of the module. The denser the void distribution, the higher the temperature of the module. The temperature of the IGBT module was reduced from 62.656 °C to 59.697 °C by using nanosilver solder paste, and the overall heat dissipation performance of the module was improved by 5%. The temperature of the module increased linearly with the increase in solder layer thickness, and the temperature increased by 0.8 °C for every 0.025 mm increase in solder layer thickness. The simulation results have a guiding significance for improving the thermal stability of IGBT modules.

## 1. Introduction

**Figure 1:**
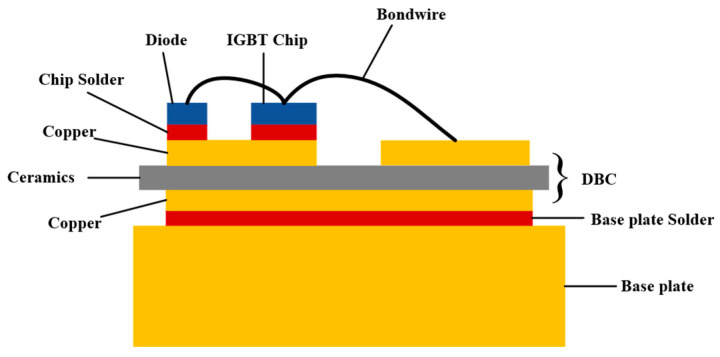
Schematic diagram of the soldering type IGBT package structure.

With the speedy development of social progress and productivity, IGBT [1] is a new variety of power electronic devices widely used in the power electronics industry. It has the advantages of high voltage resistance [2], easy driving, fast switching speed, etc., but also the advantages of a bipolar power transistor with low saturation voltage and high current handling capability. Thus, they are widely used in fields like solar energy, smart home appliances, new energy vehicles, aerospace, etc. [3,4]. At present, there are two main packaging forms for IGBT devices: soldering and crimping [5,6], of which soldering is widely used. The structure diagram of the soldering type IGBT is shown in Figure 1.

In addition to being affected by the working environment, the module will also be affected by long-term temperature changes during use. The high temperature will lead to internal fatigue aging, and the reliability [7,8,9] will be reduced. The main fatigue failure [10,11] of IGBT modules is the failure of the solder layer. The solder layer not only plays a supporting and connecting role in the IGBT module but also serves as the main heat dissipation channel. Voids are one of the main imperfections of the solder layer failure. The existence of a void greatly reduces the effective cross-sectional area for heat transfer, resulting in heat accumulation and the chip being too hot and failing. Studies have shown that the failure rate will double if the temperature of the device rises by 10 °C [12]. Due to the high cost of IGBT manufacturing and maintenance, the reliability of IGBT modules has been a hot research topic in the field of packaging. It is particularly important to extend the lifetime and improve the reliability of the module for the sustainability of power electronic systems. 

Researchers have conducted some studies on the effect of voids on IGBT modules. Haifeng Sun [13] et al. established voids at different positions in the solder layer and found that voids in the center of the solder layer have the greatest influence. Surendar [14] et al. simulated IGBT modules under CTC (conventional thermal cycling) and TSC (thermal shock cycling) impacts, and the results showed that the generation and formation of solder layer voids are accelerated. Shengxue Tang [15] et al. studied the Cauer thermal network model of void thermal resistance and its extraction method. He evaluated the degree of void growth in the solder layer by the module thermal resistance increase rate. Their research content and focus are different. However, there is no specific analysis of the influence of void formation, void position, and solder type on the temperature of the IGBT module. Since the IGBT module needs to be turned on and off continuously during operation, the heat generated by the loss can directly affect the performance of the module. Therefore, the on/off [16] state of the module was simulated in the paper.

## 2. Heat Transfer Calculation of IGBTs

### 2.1. Heat Transfer Theory

Since the IGBT will generate losses during operation, which will increase the overall module temperature, the characteristics of the material are affected by the temperature. Therefore, the current passing through the module will change, which will affect the temperature distribution in the module. According to the theory of heat transfer, the finite element of heat conduction is Equations (1) and (2) [17].

(1) $$C^{t}\overset{\cdot }{T}+K^{t}T=Q$$

(2) $$Q=Q^{nd}+Q^{c}+Q^{g}+Q^{j}$$

where $C^{t}$ is the specific heat matrix, $K^{t}$ is the heat conduction matrix, T is the node temperature vector, $\overset{\cdot }{T}$ is the temperature vector at the node that changes over time; $Q^{nd}$ is the heat flow rate vector at the node, $Q^{c}$ is convective heat vector on the surface, $Q^{g}$ is the external hate flow rate vector, $Q^{j}$ is the vector of the heat source generated by the internal heat source. Equation (3) is electrical conduction theory.

(3) $$K^{V}V=I^{nd}$$

where $K^{V}$ is the conductivity matrix, V is the node voltage, $I^{nd}$ is the node current vector. Heat generation from internal heat sources $Q^{j}$ can be obtained through the electric field Equation (4).

(4) $$Q^{j}={\int_{vol}NV_{g}}^{2}\left[\sigma (T)\right]dvol$$

where N is the shape function matrix, $V_{g}$ is the voltage gradient matrix, σ(T) is the conductivity matrix, vol is the integration variable.

**Figure 2:**
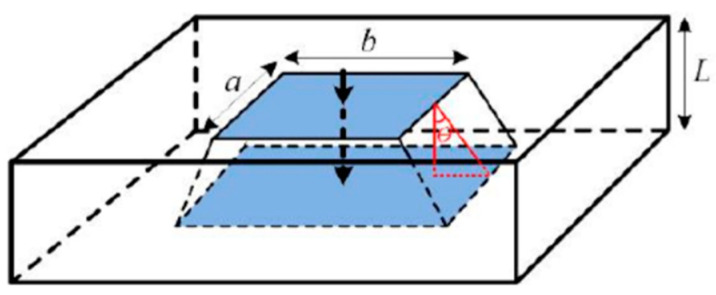
Thermal conductivity of the single-layer mechanism of the IGBT module.

Because of the special nature of the seven-layer structure inside the IGBT module, the heat diffusion angle of each layer from top to bottom of the module is not exactly the same, and the diffusion structure of each layer is shown in Figure 2. Due to the different heat transfer angles of each layer, the heat transfer area is not the same. To accurately calculate the effective heat transfer area of each layer structure, it is necessary to find out the heat transfer angle of each layer by Equations (5) and (6).

(5) $$\lambda =\frac{L}{S}$$

(6) $$f(\theta )=\left\{\begin{array}{c} 5.86\ln\lambda +40.4,\\ 46.45-6.048\cdot \lambda^{-0.969}, \end{array}\begin{array}{c} \lambda <1\\ \lambda \geq 1 \end{array}\right.$$

where S is the effective contact area of adjacent two layers, L is the thickness of each layer, and θ is the thermal diffusion angle. Therefore, the heat transfer area is given by Equation (7).

(7) $$A=\left(a+2\cdot L\cdot \tan\theta \right)\cdot \left(b+2\cdot L\cdot \tan\theta \right)$$

### 2.2. Module Loss Calculation

**Figure 3:**
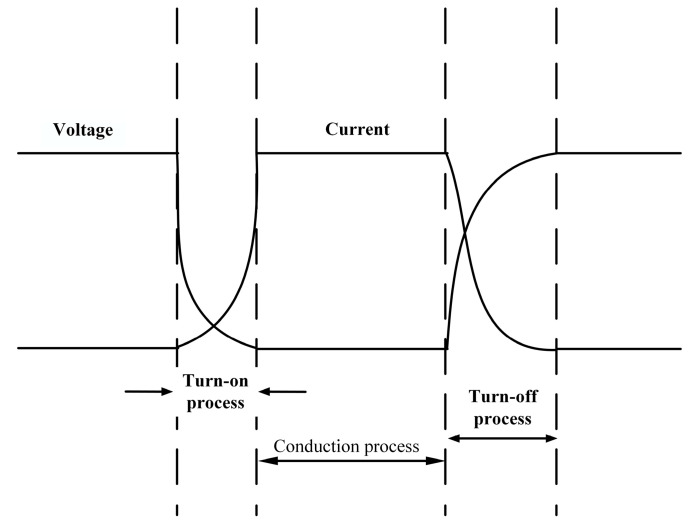
The switching process diagram of IGBT.

The loss of the IGBT module mainly includes turn-on loss, through-state loss, and turn-off loss. Figure 3 shows the process diagram of IGBT turn-on and turn-off. For the IGBT module, the main consideration is the loss of the IGBT chip and current-continuing diode.

The switching loss of the IGBT is expressed in Equation (8) [18].

(8) $$P_{sw,IGBT}=f_{sw,IGBT}\frac{1}{T_{0}}\int \begin{array}{c} T_{0}/2\\ 0 \end{array}\left(E_{on,IGBT}+E_{off,IGBT}\right)dt$$

where $P_{sw,IGBT}$ is the switching power loss of the IGBT, $f_{sw,IGBT}$ is the switching frequency, $E_{on,IGBT}$ and $E_{off,IGBT}$ are the energy losses during turn-on and turn-off, $T_{0}$ is one cycle from turn-on to turn-off.

The conduction loss is expressed in Equation (9).

(9) $$P_{{}_{cond,}IGBT}=\frac{1}{2}\left(\frac{V_{CE0}I}{\pi }+\frac{rI^{2}}{4}\right)+M\cos\varphi \left(\frac{V_{CE0}I}{8}+\frac{rI^{2}}{3\pi }\right)$$

The linearization of the output properties of the IGBTs is expressed in Equation (10).

(10) $$V_{CE\left(t\right)}=V_{CE0}+rI\sin\left(\omega t\right)$$

where $P_{{}_{cond,}IGBT}$ is the conduction power loss of the IGBT, $V_{CE0}$ is the initial on-state voltage, r is the pass-state resistance, M is the modulation index, cosφ is the power factor, φ is the phase difference between the current and voltage waves, ω is the angular frequency of the output current wave.

The switching loss of the diode is expressed in Equation (11).

(11) $$P_{sw,FWD}=\frac{1}{T_{0}}\sum_{}^{}E_{rec}\left(I_{nom},V_{nom}\right)\frac{i(t)}{I_{nom}}\cdot \frac{V_{dc}}{V_{nom}}$$

where $P_{sw,FWD}$ is the switching power loss of the diode, $E_{rec}\left(I_{nom},V_{nom}\right)$ is the diode reverse recovery energy loss, i(t)=isin(ωt) is the sinusoidal load current, $I_{nom}$ is the rated current, $V_{nom}$ is the rated voltage, and $V_{dc}$ is the DC bus voltage.

The conduction loss is expressed in Equation (12).

(12) $$P_{cond,FWD}=\frac{1}{T_{0}}\int \begin{array}{c} T_{0}/2\\ 0 \end{array}V_{CE(t)}\cdot i(t)\left[1-\tau (t)\right]dt$$

where $P_{cond,FWD}$ is the conduction power loss of the diode, $V_{CE}$ is the collector voltage, and τ(t) is the opening time.

## 3. IGBT Finite Element Model and Parameters

**Figure 4:**
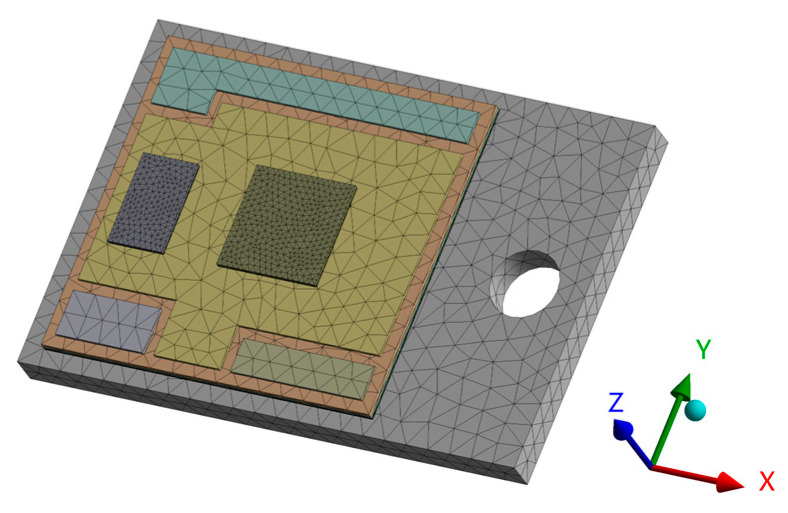
Finite element mesh model.

The IGBT module is composed of seven layers of different structures: chip, chip solder layer, DBC layer, DBC solder, substrate, thermally conductive silicone grease, and heat sink [19,20]. In this paper, a model 1200 V/50 A device from the literature [21] was used for the study. Since the device is a half-bridge structure [22] with two symmetrical diodes and chips. 1/2 of the model can be selected for the study and analysis. The mesh of the module is divided into tetrahedral cells, and the mesh of the solder layer part is carefully divided. The finite element mesh model is shown in Figure 4.

### 3.1. Model Size and Some Material Properties

The simulation process is simplified accordingly: 1. The bonding wire [23] has a small influence on the temperature of the whole module, so the bonding wire and other terminals are ignored. 2. Boundary conditions are set to simulate the heat sink. The material parameters and dimensions of each component of the IGBT module are shown in Table 1.

### 3.2. Boundary Conditions and Load Application

In the finite element thermal analysis, the chip of the IGBT is used as the main heat source. The chip is uniformly heated, and the loss of the IGBT chip is 70 W. Power loss of the chip is expressed in Equation (13).

(13) $$H=\frac{P}{V}=\frac{70w}{25.92mm^{3}}=2.7W/mm^{3}$$

H is the heat generation rate, P is the loss power of the chip, V is the volume of the chip. After calculation, the heat generation rate of IGBT is 2.7 W/mm^3^. Setting the ambient temperature at 25 °C. The convection coefficient of 4000 W/m^2^k is applied to the bottom of the substrate, and the natural convection coefficient between the three sides of the substrate and the air is 10 W·(m^2^·°C)^−1^. The IGBT module is simulated by turning on and off to increase and decrease the temperature during operation. The simulation cycle is 60 s, and the opening and closing times are both 30 s.

## 4. Analysis of the Mechanism of the Formation of Voids in the Solder Layer

Voids appear in the patch process step [25]. The patch process of the IGBT module mainly uses Sn96.5Ag3Cu0.5 solder paste (SAC305) and vacuum reflow soldering [24] process.

**Figure 5:**
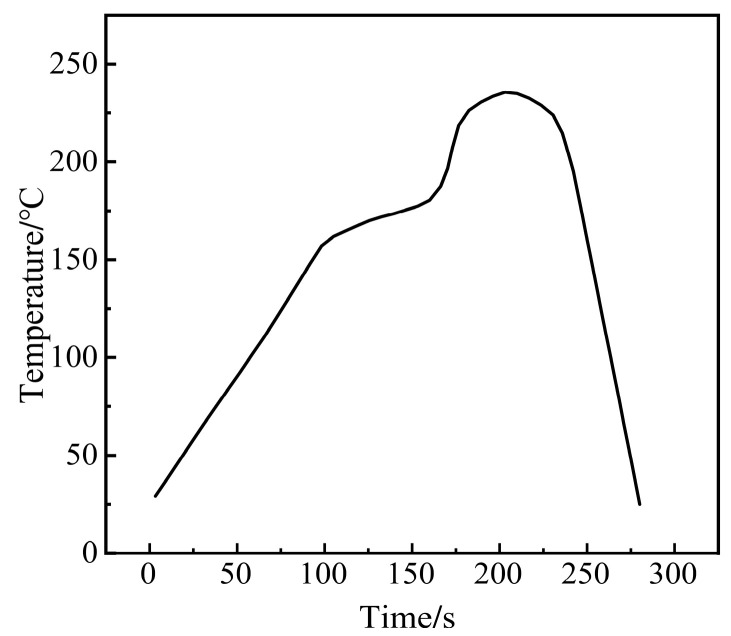
Reflow curve of SAC305 solder paste.

The melting point of SAC305 solder paste is between 217–219 °C, and the maximum peak value of reflow solder is 240 °C. It stays in the liquid phase line [26] for 60 s, and the heating coefficient [27] is 660 s °C. Therefore, the heating coefficient of the solder paste is in the best range. Figure 5 shows the corresponding reflow curve of SAC305 solder paste.

The solvent of the solder paste and some additives in the soldering process are easy to volatilize. The volatile part forms bubbles in the solder and cannot escape from the solder layer due to the substrate constraint. Incompletely volatile components tend to be trapped in the solder layer during melting and cooling, which can easily create voids. Solvents and fluxes not only have strong adhesive properties but also absorb volatiles easily and form solder joint voids. 

The process of void formation is influenced by pressure. The pressure equilibrium equation is expressed in Equation (14) [28].

(14) $$P_{i}+P_{j}=P_{s}+P_{v}+2\eta /r$$

where $P_{i}$ is the internal pressure [29] of the bubble, $P_{j}$ is the shrinkage pressure, $P_{s}$ is the hydrostatic pressure, $P_{v}$ is the pressure in the vacuum welding furnace, η is the bubble surface tension, r is the bubble radius, and 2η/r is the pressure due to surface tension. When $P_{i}+P_{j}>P_{s}+P_{v}+2\eta /r$, the bubble starts to grow bigger. When the buoyancy in the bubble can overcome the constraint of the substrate, it can move to the surface of the solder. However, this process requires a lot of energy and is not easy to control. If the energy is not enough to make the bubble buoyant, the bubble leave the substrate. As a result, the solder layer will cause voids.

### Type of Solder Layer Void

**Figure 6:**
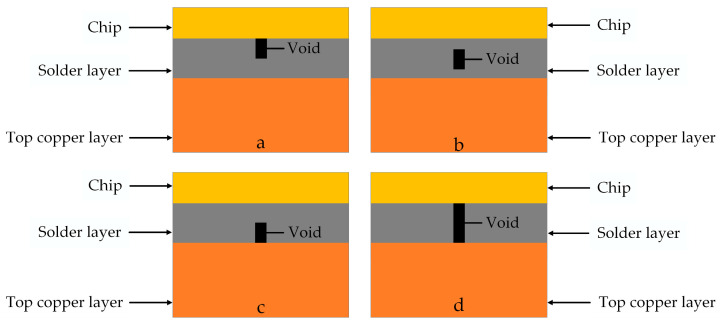
Penetrating and non-penetrating voids. (**a**) Non-penetrating void 1. (**b**) Non-penetrating void 2. (**c**) Non-penetrating void 3. (**d**) Penetrating void.

In the later simulation, a model containing a void is necessary. The shapes of voids are circular, elliptical, and irregular. The types of voids include penetration and non-penetration [12]. As shown in Figure 6, where a, b, and c are three different non-penetrating voids, and d is a penetrating void. In the literature [30], it was found that penetration voids have a greater impact on the IGBT modules. Cylindrical voids are symmetric. Thus, they are easier to model and can simplify calculations to save time. Compared to other shapes, cylindrical voids have less surface area and contact area. Therefore, they have less impact on the strength and reliability of the solder. After considering various factors, a penetrating cylindrical void was selected.

## 5. Finite Element Analysis and Discussion

### 5.1. Temperature Distribution of IGBT Module in Open-Break State

**Figure 7:**
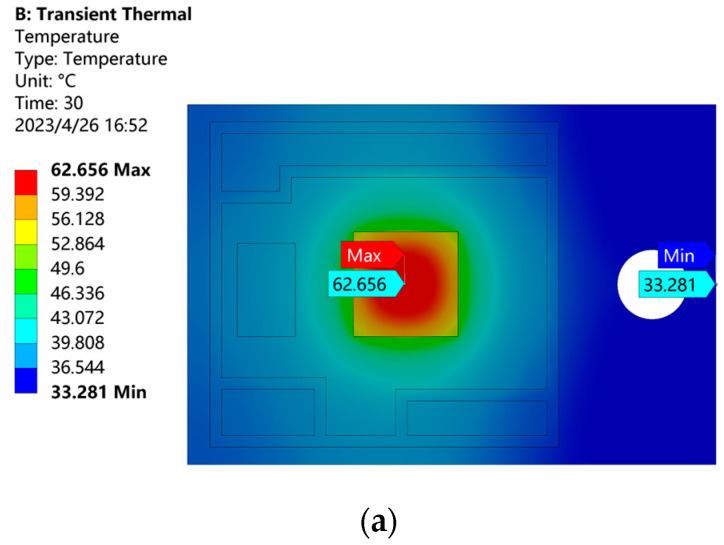

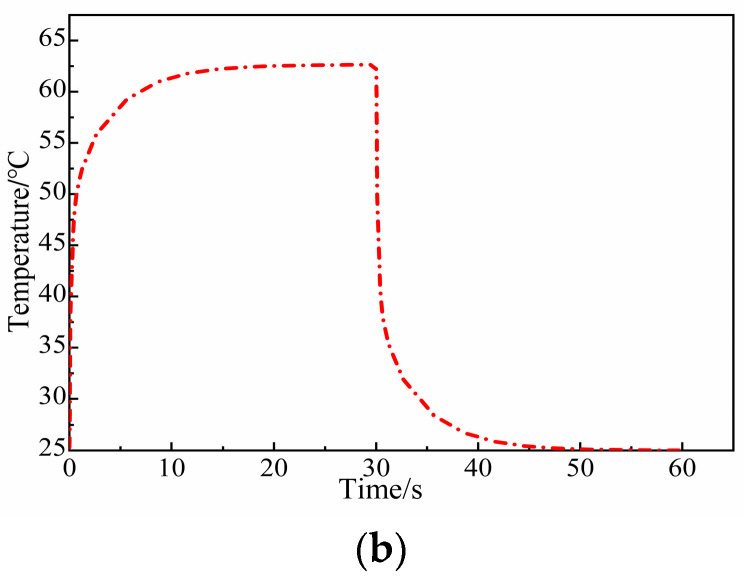
Temperature distribution of the IGBT module at 30 s and the process of temperature rise and fall. (**a**) Temperature distribution of the IGBT module at 30 s (**b**) The process of temperature rise and fall.

After transient thermal analysis, the temperature distribution cloud diagram of the IGBT module at 30 s under the loss power of 70 W is shown in Figure 7a. Due to the change of power, the internal temperature distribution of the module is not uniform, and the highest temperature is 62.656 °C in the center of the chip. Because of the thermal coupling of the module, the heat generated by the chip can only be transferred from top to bottom layer by layer. The lowest temperature at the edge of the copper substrate is 33.281 °C. As shown in Figure 7b, the junction temperature of the chip changes abruptly during the on and off operating states and gradually stabilizes to ambient temperature over time. 

### 5.2. Single Void Finite Element Analysis 

**Figure 8:**
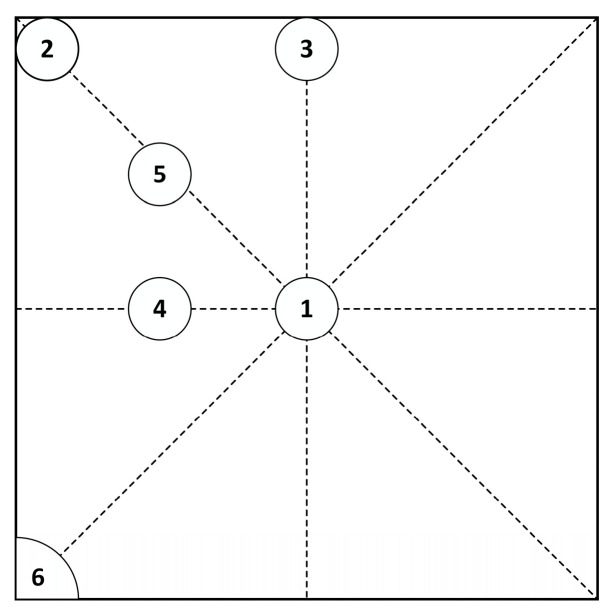
Six void positions.

Six positions in the solder layer of the chip are selected for analysis, and the radius of the void *r* = 1 mm and K = 3.9% ($K=V_{void}/V_{solder\;layer}$, K is the void rate) are taken. As shown in Figure 8, position 1 is the center of the solder layer, position 2 is at the top corner, position 3 is at the edge, position 4 is at 1/4 of the centerline, position 5 is at 1/4 of the diagonal, and position 6 appears at the tip of the chip solder layer (part of the void is inside the solder layer). The effect of void damage at different positions on the junction temperature of the module is studied by transient thermal analysis.

Fourier’s law of heat conduction can be expressed in Equations (15) and (16).

(15) Q=−λA∇T

(16) $$\nabla T=-\frac{S\cdot H}{\lambda \cdot C}$$

where Q denotes the total heat flow during thermal conduction, λ denotes the thermal conductivity, ∇T denotes temperature gradient, A denotes the vertical cross-sectional area of heat flow through. S is the area of the void, H is the power loss of the chip, C is the circumference of the void. “-” indicates that the direction of heat transfer is opposite to the direction of the temperature gradient.

**Figure 9:**
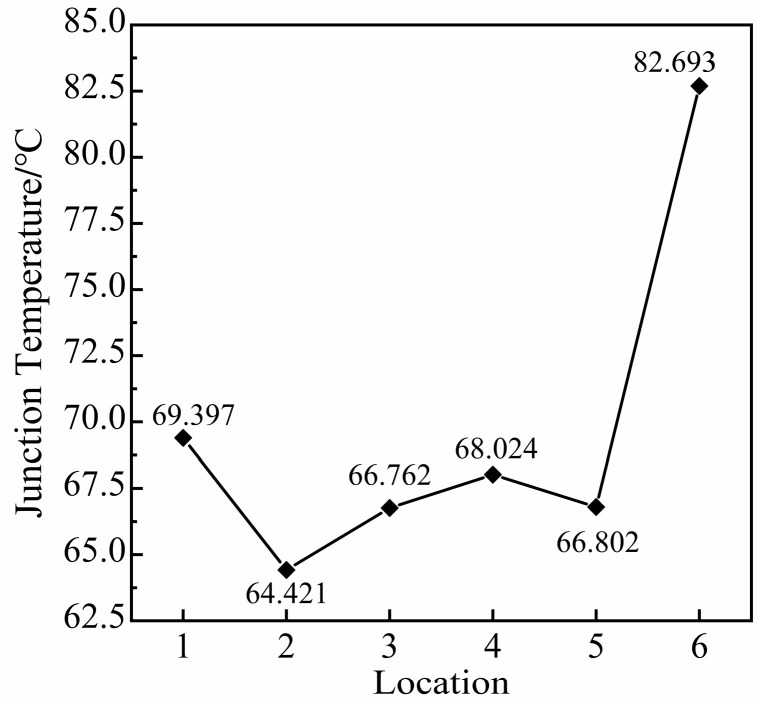
Relationship between void positions and junction temperature.

As can be seen from Figure 9, positions 1–5 are complete voids. From the law of heat transfer: the ratio of H/λ is kept constant, and the effect on the junction temperature depends on the ratio of S/C. As the ratio of S/C increases, the temperature gradient ∇T perpendicular to the chip also increases, which eventually leads to the temperature of the chip increases. The closer the void is to the center of the solder layer, the larger the temperature gradient ∇T perpendicular to the direction of the chip.

The area and circumference of the void can be expressed in Equations (17) and (18). Equation (19) can be deduced from Equation (16).

(17) $$S=\pi r^{2}$$

(18) C=2πr

(19) $$\nabla T=-\frac{S\cdot H}{\lambda \cdot C}=-\frac{r}{2\lambda }H$$

where r is the void radius. Additionally, the position 6 part of the void has been beyond the solder layer. The void is 1/4 cylindrical. From Equation (19), the heat generated above the void is proportional to the radius of the void. The heat production increases with the increase in the void radius. Thus, the temperature field distribution changes greatly, and the junction temperature rises sharply. To keep the same void rate, the radius of the void at position 6 is twice as large as the radius of the complete type void. When the void is at position 6, the chip has the highest temperature. Therefore, the radius of the void affects the junction temperature more than many other factors. 

Void generation is difficult to avoid. On the one hand, it is necessary to avoid the void at the edges and top corners of the solder layer during the device production process. The specific shape and radius of the void have a significant effect on the junction temperature. On the other hand, when the same void rate is maintained inside the soldering layer. The closer the void is to the center of the solder layer, the higher the module’s temperature. 

### 5.3. Multiple Voids Finite Element Analysis

**Figure 10:**
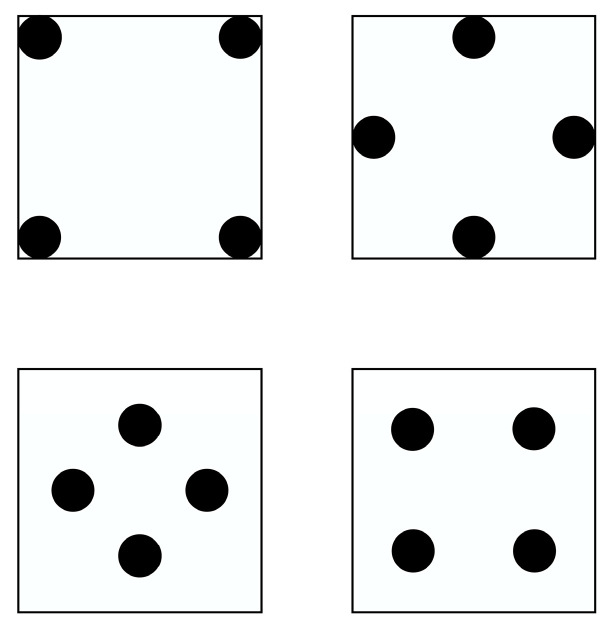
Distribution of multiple voids.

The solder layer voids will change during production or use. The changed voids may appear as single larger voids or random and uneven voids. As shown in Figure 10, multiple voids are distributed at the top corner, edge, 1/4 of the diagonal, and 1/4 of the center line of the solder layer. The influence of the position distribution and size change of multiple voids on the maximum temperature of modules are analyzed.

This simulation uses five sets of data (K = 3.9%, 7.6%, 15.5%, 26.2%, 34.9%; K is the void rate) to analyze the effect of multiple voids’ position and size on the module’s junction temperature.

**Figure 11:**
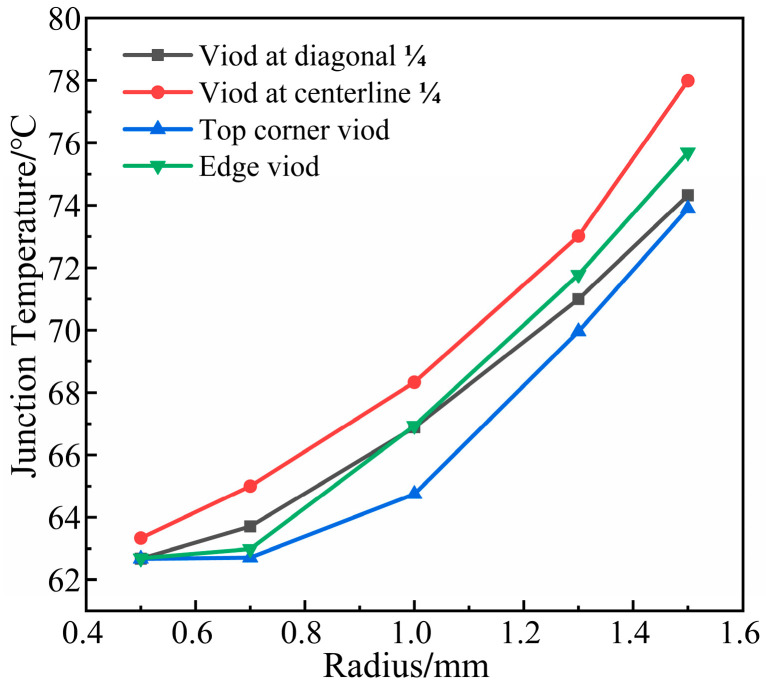
Relationship between multiple voids radius and junction temperature.

As shown in Figure 11, when the radius of the void < 0.7 mm, the temperature of the module rises gently. After the radius of the void > 0.7 mm, the module’s temperature rises steeply. It was found that when the void rate of the solder layer was greater than 5% in the literature [31] experiment, IGBT modules began to enter the failure state, causing the characteristics of the module to change. A study [32] tested the reliability of IGBT devices with different void rates through power cycle experiments and found that when the void rate is greater than 5%, it can cause device failure directly. The void control standard of the loading process is established through experiments. The single void rate is less than 2%, and the total void rate of multiple voids is less than 5%. When the radius of void = 0.7 mm (k = 7.6%), the module began to fail. Therefore, 0.7 mm is the special point of these five sets of data. As the void rate increases, the solder layer material is replaced by internal air with lower thermal conductivity, which reduces the heat dissipation capability. Voids in ¼ of the center line have the highest module temperature. Due to the relatively concentrated distribution of the void and close to the center of the solder layer, more heat is prevented from conducting downward, accelerating the accumulation of heat and making the temperature of the module maximum. The top corner voids are far from the center of the solder layer and distribute sparsely, which present less obstruction to heat transfer. Thus, the module has the lowest temperature.

### 5.4. Multiple Voids Spacing Finite Element Analysis 

**Figure 12:**
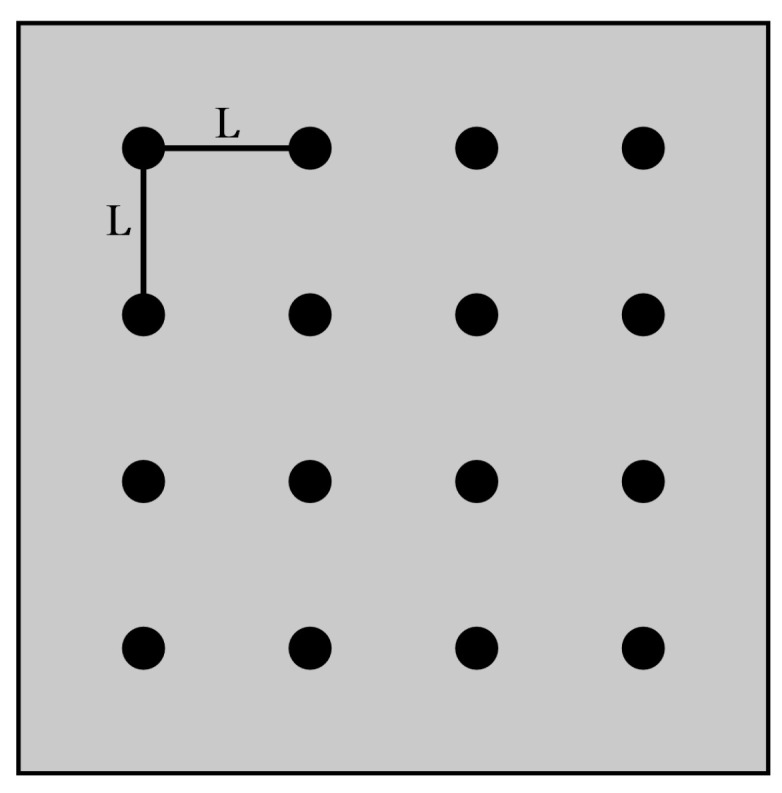
Distribution of void spacing.

As shown in Figure 12: 16 voids are selected in the solder layer, and the radius of each void is 0.5 mm (total void rate is 15.5%). The voids are distributed evenly. The void spacing is taken as 0.2 mm, 0.4 mm, 0.6 mm, 0.8 mm, and 1.0 mm. The influence of the distance between voids on the junction temperature of modules is analyzed.

**Figure 13:**
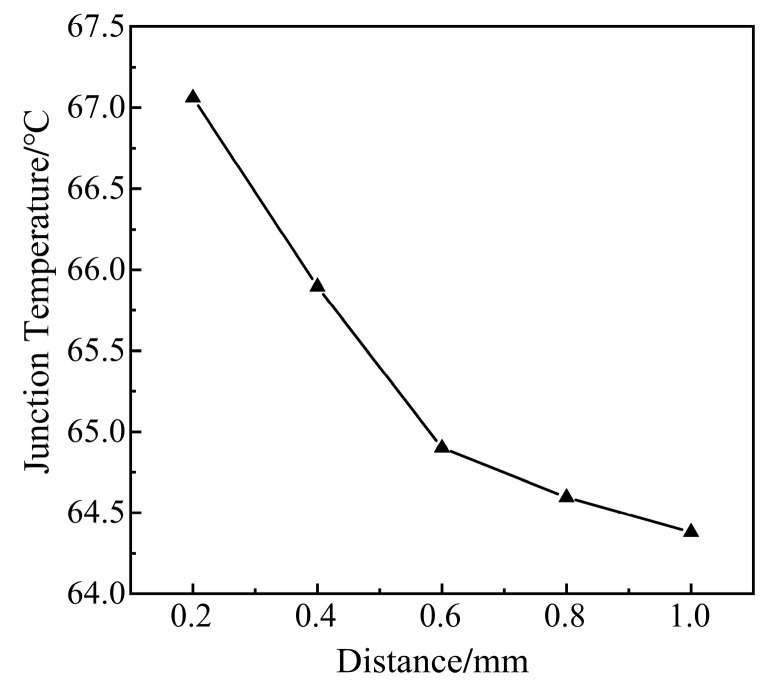
Relationship between void spacing and junction temperature.

As shown in Figure 13, the void spacing is inversely related to the junction temperature of the module. The smaller the void spacing, the higher the temperature of the module. 

When the solder layer is without voids, the heat generated by the chip will pass through the horizontal and vertical directions. Voids in the solder layer will block the vertical heat transfer path above the chip, and the heat dissipation efficiency of the module will be reduced as a result.

The spacing of the void is small enough, and the surrounding voids can merge and connect to form a larger void easily. The larger the void, the smaller the cross-sectional area of heat conduction. From Equation (15), when the heat flux Q is constant, the cross-sectional area A of heat passing through is smaller. The larger the temperature change rate in the direction perpendicular to a given section, the larger the temperature gradient ∇T on the lower surface of the chip. When the temperature gradient ∇T increases, the temperature of the module will also increase. With the increase in void spacing, the junction temperature of the module tends to be stable. The scattered voids can form individual temperature centers above the chip, dispersing the heat buildup. This means that dispersed multiple voids have a weaker effect than a single large void.

### 5.5. Effect of Solder Layer Voids on Module Junction Temperature under Different Materials

Studies showed that nanosilver solder paste had better creep resistance and shear stress than solder alloys. Hensler et al. [33] found that the service life of the nanosilver solder paste connection module was six times longer than the solder alloy through temperature cycling tests. Chen [34] et al. found that sintered nanosilver joints have better resistance to shear stress and cyclic loading than SAC305 in high-temperature environments. Thus, nanosilver joints have longer fatigue life. Related studies [35,36,37] showed that the resistivity and thermal conductivity of nanosilver solder pastes have better performance compared to other solders, which makes the module generate less heat. Thus, nanosilver solder paste can enhance the heat dissipation efficiency of the module and adapt to high temperatures.

**Figure 14:**
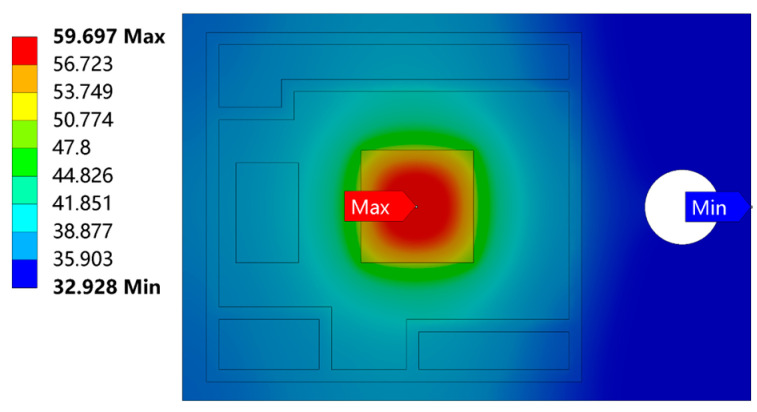
Temperature of the module when nanosilver is the solder paste.

As shown in Figure 14, the solder layer is in an ideal condition without damage. When nano silver is the solder paste, the maximum temperature of the module is 59.697 °C. Compared with Figure 7a, the temperature decreases from the original 62.656 °C to 59.697 °C. When nano silver solder paste is used, the temperature of the module is reduced by 2.959 °C, and the heat dissipation rate is increased by about 5%.

**Figure 15:**
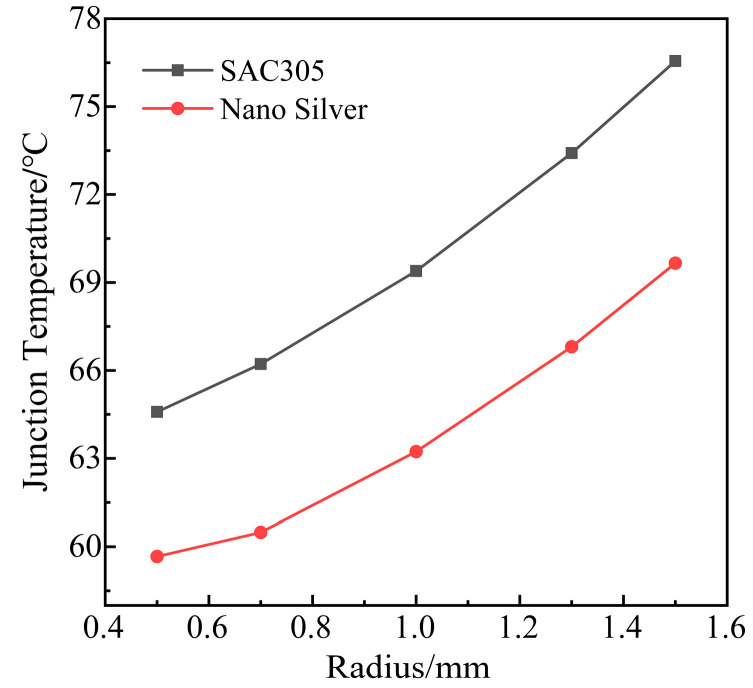
Temperature comparison between SAC305 solder and nano silver solder paste.

From Figure 15, with the increase in the void, the temperature of the nanosilver solder module is lower than that of the SAC305 solder module. Nanosilver solder paste has more obvious advantages. The solder layer void radius is 1.5 mm, and the maximum temperature of the module is reduced by about 7 °C with the nanosilver solder paste. The thermal conductivity of nanosilver solder paste is 240 W/m·K, and the thermal conductivity of SAC305 solder is 32.7 W/m·K. The thermal conductivity of the former is much greater than that of the latter. Therefore, nanosilver as solder paste can make IGBT module heat dissipation quickly, which can further improve the thermal performance and reliability of the module.

Thus, with or without voids, the nanosilver solder paste can demonstrate better thermal stability and reduce the temperature of the module. However, the cost of high thermal conductivity materials is high. The industry needs to make a balance between cost and performance according to the actual needs.

### 5.6. Effect of Solder Layer Thickness on Temperature Distribution in the Presence of Voids

Referring to the chip placement soldering process of the IGBT module, the thickness of the solder layer is analyzed in 0.1~0.2 mm for parameters, and the step is 0.025 mm. The void is located in the center of the solder layer with a radius of 1 mm. Varying the thickness of the solder layer to analyze the change in the module’s temperature.

**Figure 16:**
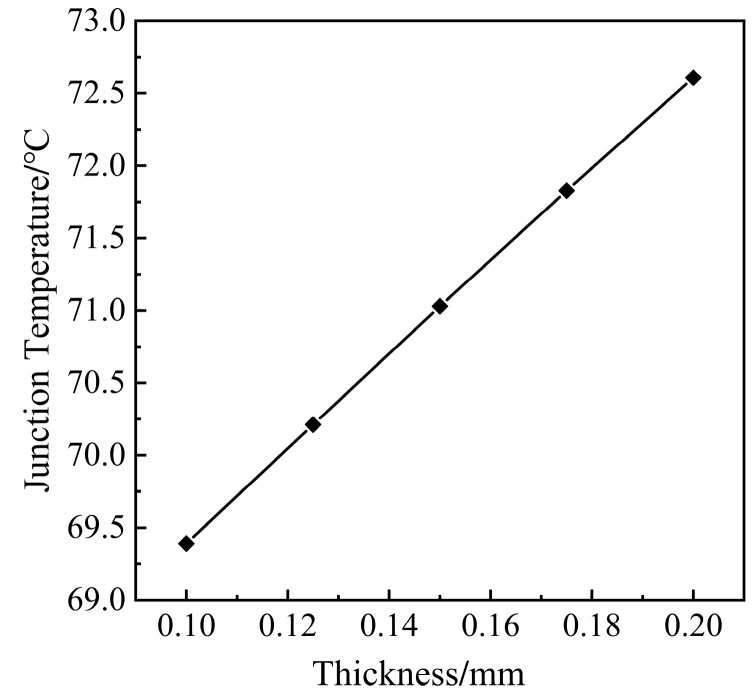
Relationship between the thickness of the solder layer and junction temperature.

From Figure 16, the thickness of the solder layer increases almost linearly with the maximum temperature of the module. The thickness of the solder layer increases by 0.025 mm, and the temperature of the module increases by about 0.8 °C.

According to the heat transfer theory, the thermal resistance of the IGBT module is expressed in Equation (20) [38] and Equation (21) [39].

(20) $$R_{th}=\frac{L}{kA}$$

(21) $$T_{j}=T_{a}+R_{th\left(j-a\right)}\cdot P_{loss}$$

where $R_{th}$ is the thermal resistance of the solder layer, L is the thickness of the solder layer, k is the thermal conductivity, A is the solder layer heat sink area. $T_{j}$ is the module junction temperature, $T_{a}$ is the ambient temperature, $R_{th\left(j-a\right)}$ is the thermal resistance between junction temperature—ambient temperature, $P_{loss}$ is the power loss of the chip. As L increases, $R_{th}$ also becomes larger, resulting in the accumulation of heat during the transfer process and causing the temperature of the entire module to rise eventually. Therefore, the junction temperature of the module can be controlled by thinning the thickness of the solder layer. 

## 6. Conclusions

In this paper, the effect of solder layer void damage on the temperature of the IGBT module is studied by finite element simulation. The results indicate that the temperature of the IGBT module increases linearly with the increasing void ratio of the chip solder layer. The void at the top corner of the solder layer has the greatest impact on the junction temperature of the module. Therefore, the thermal stability can be improved effectively by using the technology to avoid voids occurring at the top corner of the solder layer. The shape of the void is also one of the factors affecting the temperature of the module. The smaller the void spacing and the denser the distribution, the higher the temperature of the module. Compared with SAC305 solder paste, nanosilver solder paste has better thermal conductivity and service life, which can improve the thermal stability and reliability of the module effectively. The heat dissipation of the module has been improved by about 5%. The thickness of the solder layer can also affect the temperature distribution of the whole module. The temperature of the module grows linearly with the thickness of the solder layer, and the temperature increases by 0.8 °C with increasing the thickness of the solder layer by 0.025 mm. Therefore, we need to choose the right thickness of the solder layer to further improve the reliability of the IGBT module. 

## Figures and Tables

**Table 1:** Material parameters [21,24] and dimensions of each component of the IGBT module.

Components	Material	Thermal Conductivity /(W·m^−1^·K^−1^)	Specific Heat Capacity /(J·Kg^−1^·K^−1^)	Density /(Kg·m^−3^)	Length /(mm)	Width /(mm)	Thickness /(mm)
IGBT chip	Si	149	700	2300	9	9	0.32
Diode	Si	149	700	2300	5	8	0.30
Chip Solder Layer	SAC305	32.7	150	7500	9	9	0.10
DBC	Cu	401	380	8900			0.30
	Al_2_O_3_	35	600	3600	30	28	0.40
	Cu	401	380	8900	30	28	0.30
Substrate solder layer	SAC305	32.7	150	7500	30	28	0.20
Substrate	Cu	401	380	8900	45.5	31	3.00

## Data Availability

Not applicable.
